# Sharing in Caring: Family Caregiving Task-Sharing Patterns for Older Adults in Singapore

**DOI:** 10.1093/geronb/gbae186

**Published:** 2024-11-13

**Authors:** Jeremy Lim-Soh, Pildoo Sung, Ha-Linh Quach, Rahul Malhotra

**Affiliations:** Centre for Ageing Research and Education, Duke-NUS Medical School, National University of Singapore, Singapore, Singapore; Department of Sociology, Hanyang University, Seoul, South Korea; Centre for Ageing Research and Education, Duke-NUS Medical School, National University of Singapore, Singapore, Singapore; Centre for Ageing Research and Education, Duke-NUS Medical School, National University of Singapore, Singapore, Singapore; Health Services and Systems Research, Duke-NUS Medical School, National University of Singapore , Singapore, Singapore

**Keywords:** Activities of daily living, Health and social services, Latent class analysis, Long-term Care, Socioemotional needs

## Abstract

**Objectives:**

Research on family caregiving for older adults has largely focused on primary caregivers. We identify caregiving task-sharing patterns among multiple caregivers, including family members and live-in hired workers. In addition, we investigate caregiver and care-recipient characteristics associated with these patterns.

**Methods:**

We interviewed 278 primary family caregivers of home-dwelling older adults in Singapore about who provides what assistance across 3 domains: activities of daily living, health and social services use, and socioemotional and other needs. Latent class analysis was used to identify caregiving task-sharing patterns.

**Results:**

Three patterns were identified: (a) “Shared-Diverse” (39%)—multiple caregivers assisting in all 3 domains, (b) “Shared-Domestic” (32%)—multiple caregivers assisting with activities of daily living and socioemotional and other needs, and (c) “Solo-Diverse” (29%)—a sole caregiver assisting in all 3 domains. “Solo-Diverse” caregivers were less likely to be employed and had higher depressive symptoms relative to “Shared-Diverse” primary family caregivers.

**Discussion:**

The predominance of caregiving task-sharing patterns involving multiple caregivers calls for expansion of research, policies, and programs beyond primary caregivers. Greater attention should be given to how families and live-in hired workers share caregiving tasks for older adults, and how this varies across cultural settings, especially in familial Asian societies. The findings further highlight the vulnerabilities of solo caregivers, whose employment capacity and mental health may be adversely affected by their caregiving duties. Policy-makers should ensure that solo caregivers have access to support programs that address their financial and mental health needs.

Family caregivers play a critical role in meeting the needs of many older adults with long-term care needs, including functional limitations, chronic illnesses, and cognitive disabilities (Schulz et al., 2020). They perform diverse tasks, including assistance with activities of daily living (ADLs) and instrumental ADLs (IADLs), facilitating older adults’ access to health and social services, and providing socioemotional support. Family caregivers’ contributions are even more important in societies where the state provides limited institutional care for the aging population, which is often the case in developing countries and countries with more residual welfare systems (Verbakel, 2018).

A growing body of research thus focuses on caregiving tasks and their implications for the health and well-being of family caregivers. However, both theoretical and empirical research works have mainly focused on the role of the primary family caregiver, underexplored the possibility that more than one individual is actively sharing caregiving tasks (Ellis et al., 2023; Hu et al., 2023; Marcum et al., 2020). Less is known about (a) how the different types of caregiving tasks are distributed among primary and secondary caregivers, (b) which primary family caregiver and care-recipient characteristics are associated with the distribution of caregiving tasks, and (c) whether sharing caregiving tasks influences the mental well-being of primary family caregivers.

By addressing these gaps, this study aims to provide a deeper understanding of how family caregiving is distributed across multiple caregivers, which will be instrumental in designing better policies to support caregivers. In addition, identifying which older adult and primary family caregiver characteristics are associated with receiving help from multiple caregivers (vs only one) can facilitate more targeted interventions (Hu et al., 2023). Considering that support from secondary caregivers may alleviate caregiver burden (Liang et al., 2022), testing for associations between identified caregiving patterns and caregiver depressive symptoms further helps pinpoint psychologically vulnerable groups of primary family caregivers.

This study was conducted in Singapore, which provides an interesting setting to investigate the topic of shared care because its small geographical size contributes to the potential for extended family members to live in proximity, facilitating multiple care exchanges. Furthermore, it is common in Singapore to employ live-in migrant domestic workers (MDWs) to assist with caregiving and other household tasks (Yeoh & Huang, 2009), which results in a further division of caregiving tasks.

## Literature Review

### Caregiving Tasks and How They Are Shared

The tasks that caregivers undertake depend on the care needs of older adults, as well as the ability and willingness of caregivers to meet those needs (Curnow et al., 2021). According to a recent review of family caregiving for older adults, caregiving tasks may include assistance with household tasks, self-care tasks, mobility, provision of emotional support, maintaining social connections, health and medical care, advocacy and care coordination, and surrogacy (Schulz et al., 2020).

Most caregivers engage in a selection of these potential tasks. For instance, Huang et al. (2021) identified five distinct caregiving patterns: All-Round Care (High Demand, 19.5%), All-Round Care (Moderate Demand, 8.2%), Predominant IADLs Care (High Demand, 23.8%), Predominant IADLs Care (Moderate Demand, 32.5%), and Minimal ADLs and IADLs Care (Low Demand, 16.0%). However, their study focused only on the care provided by one caregiver and did not explore the role of shared caregiving.

Some indication of how caregiving tasks are shared can be elicited from caregivers’ self-identification of their caregiving roles. In the United States, 45% of caregivers see themselves as the sole caregiver, 17% as the primary caregiver contributing the most, 24% as a nonprimary caregiver contributing less, and 13% as a nonprimary caregiver contributing equal to a primary caregiver (NAC & AARP, 2020). Task sharing can also be related to care needs; for example, older adults with dementia are likely to have larger caregiving networks (Spillman et al., 2020).

One study considered the caregiving tasks of both primary and secondary caregivers; however, the primary and secondary caregivers’ responses were treated as separate observations without analyzing how caregiving tasks were shared between them (Ali et al., 2022). Another recent study measured the difference in hours of care and the number of activities between primary and secondary caregivers; however, the analysis did not address types of caregiving tasks (Hu et al., 2023). Therefore, a gap remains in the literature regarding how different caregiving tasks are shared among multiple caregivers.

### Caregiver and Care-Recipient Characteristics Related to the Sharing of Caregiving Tasks

It is not surprising that care-recipient characteristics dictate the types of care required, as well as how they are shared. Older care recipients and those with more functional limitations are likely to need extensive assistance with ADLs and IADLs, potentially leading family members to hire an MDW. Care recipients with chronic conditions may require assistance to attend regular health checkups. Care recipients who receive community care services, such as spending time at a daycare center, may also need assistance with transportation. Care recipients with cognitive impairment or dementia typically have complex care demands, often receiving help from multiple caregivers (Ali et al., 2022; Ellis et al., 2023).

On the other hand, the role of caregiver characteristics in caregiving patterns is less straightforward. Although younger caregivers, such as children of the care recipient, may have a greater ability to provide physical care, they could also have other work and family responsibilities (Ali et al., 2022). Gender impacts the division of caregiving tasks; for example, women are frequently viewed as family “kin-keepers” who can support the socioemotional ties of a spouse or parent, especially one who is depressed (Ang, 2019; Lim-Soh et al., 2024). Other studies find that women are more likely to provide instrumental care for ADLs and IADLs (Liu et al., 2017). Furthermore, female caregivers are less likely to be employed than noncaregivers (Lee & Tang, 2015), due to the role strain of balancing work and caregiving responsibilities (Edwards et al., 2002). Lastly, due to low fertility and small family sizes, some caregivers may not have siblings who can help to share caregiving tasks, resulting in solo caregiving patterns (Tan, 2022).

### Caregiving Tasks and Mental Well-Being

Family caregivers often face stress and threats to mental health. According to the stress process model, caregivers face primary stressors resulting from the care needs and health conditions of the care recipient, including cognitive impairment, problematic behavior, and functional limitations, as well as secondary stressors such as family conflict, economic strain, and the loss of social activities (Pearlin et al., 1990). The stress of caregiving is compounded by the protracted and uncertain nature of the role, as one never knows when the care recipient’s condition will deteriorate, leading to chronic stress exposure (Schulz et al., 2020). Ultimately, this makes caregiving a mental health risk (Park, 2021; Schulz & Sherwood, 2008). For example, in an American study utilizing panel data, older adults who became caregivers for their spouses during the study duration had higher depressive symptoms than noncaregivers (Dunkle et al., 2014).

Although the general stress of caregiving is well documented, it is only recently that caregiving tasks have received attention within the stress process model (Liu et al., 2017). For example, while providing instrumental care can worsen depression, emotional care can have the opposite effect (Liu et al., 2017). In addition, family caregivers’ mental health may be related to their employment status; caregivers who simultaneously work may be exposed to role strain, or difficulty meeting the dual demands imposed on them (Edwards et al., 2002). On the other hand, sharing caregiving tasks with a secondary caregiver or domestic helper may lessen caregiver burden and distress (Chong et al., 2014; Liang et al., 2022; Østbye et al., 2013). However, while sharing responsibilities can alleviate the load, poor coordination among multiple caregivers is associated with increased caregiver depressive symptoms (Xu et al., 2021). Therefore, it is crucial to explore the association of the sharing of caregiving tasks with caregivers’ mental health, especially that of primary caregivers who often bear the largest burden.

## The Present Study

The objectives of this study are threefold: (a) to identify prevalent caregiving task-sharing patterns, based on how different caregiving tasks are shared between primary and secondary caregivers, (b) to examine primary family caregiver and care-recipient characteristics associated with the identified patterns, and (c) to explore the association of the identified patterns with primary family caregiver depressive symptoms. To address these aims, we consider 3 types of assistance care recipients receive (with ADLs and IADLs, health and social care services use, and socioemotional and other needs) from multiple caregivers and apply latent class analysis (LCA) to survey data on 278 home-dwelling older adults aged 75 years and older in Singapore.

Families have traditionally been at the forefront of caring for older adults in Singapore. The state provides means-tested assistance to those without kin, often in partnership with voluntary welfare organizations or charities (Mehta, 2006). Over the past decade, eldercare policies have evolved, with a nascent universal long-term care insurance scheme (Fong & Borowski, 2022), and an expanded public health system at the community level (Chan, 2021). Nevertheless, family caregivers remain central to the eldercare ecosystem in Singapore, not just to provide direct assistance, but also to help older adults navigate the new and complex services that they can access.

With a population consisting of ethnic Chinese, Malays, and Indians, Singaporean society is also historically influenced by familism-oriented Asian cultural norms, such as Confucianism (Yeh, 2022). A significant majority (73%) of Singaporeans believe that the family has the primary responsibility for caring for older adults, ahead of the state and the community (Gee et al., 2018). Older adults often co-reside with children and grandchildren in multi-generational households, or at least live in close proximity (Lim-Soh, Tan, et al., 2023). Notwithstanding the changing preferences of both younger and older generations, these trends are cemented by housing policies that encourage adult children to live with or near their parents, which facilitates family caregiving (Narayanankutty & Dommaraju, 2023).

Caregivers are often not alone in Singapore. There is a cultural expectation for extended family members to contribute to caregiving, which can lead to disappointment if not met (Basnyat & Chang, 2021). It is also common for Singaporean families to employ an MDW, or full-time live-in helper, who assists with the housework as well as caregiving duties, particularly for labor-intensive activities (Yeoh & Huang, 2009). The involvement of MDWs in Singapore contributes additional dynamics in how caregiving tasks are distributed among multiple caregivers, including family members and MDWs (Azman et al., 2024).

## Method

### Sample

This study utilized data from the baseline wave of the Caregiving Transitions among Family Caregivers of Elderly Singaporeans (TraCE) survey, conducted from 2019 to 2020 (Lim-Soh, Azman, et al., 2023). Singaporeans aged 75 years and above who had previously participated in nationally representative surveys (Chan et al., 2018, 2019) were screened for the receipt of human assistance for ADLs and IADLs and identified as care recipients. Of the 395 eligible care recipients, 278 (70%) were recruited in the baseline survey along with their primary family caregivers.

A primary family caregiver was defined as a family member aged 21 years and above who fulfilled at least two of the following criteria: (a) was the person most involved in providing direct care for day-to-day activities, (b) was the person most involved in ensuring provision of care, and (3) was the person most actively participating in making decisions for care and treatment. MDWs were not eligible to be selected as primary family caregivers due to their contractual employment relationship with the care recipient and their family. The primary family caregiver provided proxy responses if the care recipient was unable to respond for health reasons. Respondents provided written consent, and the study was approved by the Institutional Review Board of the National University of Singapore (LS-18-387C).

### Measures

#### Caregiving tasks

The primary family caregiver was asked for how many hours in a typical week he/she assisted the care recipient with (a) ADLs and IADLs (ADLs: take a bath/shower, dress up, walk around the house, stand up from or sit down on a bed/chair, use the sitting toilet, eat; IADLs: take public transport to leave home, leave the home to purchase necessary items or medication, take care of financial matters such as paying utilities, dust, clean-up and other light housework, prepare own meals, take medication as prescribed, use the phone), (b) healthcare or social care services use (e.g., accompanying care recipient to the doctor, setting up and changing medical appointments, bringing care recipient to and from the older adult care center), and (c) socioemotional and other needs (e.g., personal supervision of care recipient, helping care recipient to read and communicate with others, providing emotional support such as listening to and comforting care recipient). The responses were recoded to reflect whether the primary family caregiver helped with each type of activity (0 = did not help, 1 = helped).

The primary family caregiver was also asked whether anyone else (including other family members, friends, and neighbors, MDWs, paid workers, visiting nurses) helped with each of these activities, and if so, for how many hours. Other family members were identified as secondary caregivers in this analysis, and so were MDWs because the live-in nature of their work greatly affects family caregiving task-sharing patterns (Azman et al., 2024; Chong et al., 2014). Help from friends and neighbors was not reported by any respondent, and four formal caregivers—two part-time cleaners, a nurse, and a doctor—were excluded from this analysis on family caregiving. The responses were then recoded to reflect whether any secondary caregiver helped with each type of activity (0 = did not help, 1 = helped).

#### Participants’ characteristics

We collected data on the following care recipients’ characteristics: age, gender, marital status (0 = single/widowed/divorced, 1 = married), education (1 = no formal education, 2 = primary, 3 = secondary, 4 = vocational, 5 = junior college/polytechnic, 6 = university), number of chronic conditions, number of ADL and IADL limitations, dementia symptoms reported by the primary family caregiver (Eight-item Informant Interview to Differentiate Aging and Dementia—AD8; range from 0 to 8; Cronbach’s alpha = 0.88) (Galvin et al., 2005), and whether they used community care services (such as home care and daycare centers).

We also considered the following primary family caregivers’ characteristics: age, gender, marital status, education, employment status (0 = not working, 1 = working), whether the primary family caregiver co-resided with the care recipient, how many siblings they had, and depressive symptoms (Center for Epidemiological Studies Depression scale—CES-D; range from 0 to 22; Cronbach’s alpha = 0.83; Kohout et al., 1993). We also recorded whether the primary family caregiver was the child of the care recipient, as this is the predominant family caregiving relationship in Singapore (Chan et al., 2011).

Lastly, we accounted for the following caregiving-context characteristics: whether an MDW helped with caregiving and the total number of secondary caregivers (including MDWs).

### Analysis

Following recent studies on caregiving tasks (Ali et al., 2022; Huang et al., 2021), LCA was applied to identify family caregiving task-sharing patterns and their distribution between primary family caregivers and secondary caregivers. LCA is commonly referred to as a person-centric method because it describes differences between people across a set of multiple indicators (Lanza, 2016). In this study, it might be more appropriate to call it family-centric, as the indicators used apply to the older care recipient as well as their multiple caregivers.

We used a total of six indicators across two dimensions: three types of caregiving tasks and the identity of the caregiver (primary or secondary). The indicators used in LCA were dichotomized to facilitate clearer identification of patterns across the two dimensions (whether primary and secondary caregivers engaged in different types of caregiving tasks). Class enumeration was conducted using the R package “MplusAutomation” to run multiple LCA models and compare their goodness-of-fit (Supplementary Table 1). Various fit statistics were consulted including the Akaike information criterion (AIC), adjusted Bayesian information criterion (BIC), Vuong–Lo-Mendell–Rubin adjusted likelihood ratio test *p*-value (VLMR), bootstrapped likelihood ratio test *p*-value (BLRT), lowest average latent class posterior probability, and approximate correct model probability (cmP; Nylund-Gibson & Choi, 2018). LCA uses full information maximum likelihood estimation, such that patterns were identified for respondents who did not respond to all questions on caregiving tasks (see Table 1 for details); however, one respondent was excluded from the analysis due to missing responses on all questions.

**Table 1.** Characteristics of Care Recipients and Their Primary Family Caregivers (*N* = 278)

Variable	Observations	%	Mean (*SD*)
*Types of caregiving tasks*			
Primary family caregiver helps with			
ADLs and IADLs	268	85.8	
Health and social services use	256	67.6	
Socioemotional and other needs	263	89.4	
Secondary caregivers help with			
ADLs and IADLs	270	64.8	
Health and social services use	272	33.8	
Socioemotional and other needs	269	49.1	
*Care-recipient characteristics*			
Age, in years [range = 75–104]	278		85.4 (5.4)
Male	278	33.5	
Married, currently	278	40.6	
Education	278		1.7 (1.0)
Chronic conditions	278		4.6 (2.5)
ADL limitations	278		2.3 (2.4)
IADL limitations	278		3.9 (2.0)
AD8 score	278		3.6 (2.9)
Use of community care services	278	31.7	
*Primary family caregiver’s characteristics*			
Age, in years [range = 23–90]	278		61.7 (12.0)
Male	278	26.6	
Married, currently	278	60.4	
Education	278		3.3 (1.5)
Employment status	277	41.9	
Child of care recipient	278	73.0	
Co-residing with care recipient	278	86.3	
Number of siblings	278		3.5 (2.2)
CES-D	278		4.6 (4.1)
*Caregiving context*			
Migrant domestic worker helps with caregiving	278	48.2	
Total informal caregivers	278		2.3 (1.2)

*Notes*: AD8 = Eight-item Informant Interview to Differentiate Aging and Dementia; ADLs = activities of daily living; CES-D = Center for Epidemiologic Studies Depression scale; IADLs = Instrumental ADLs; *SD* = standard deviation.

To describe differences between the identified patterns, characteristics of care recipients and primary family caregivers were reported for each pattern. We also tested significant differences between the patterns using analysis of variance (ANOVA) for continuous variables and the chi-square test for categorical variables. In addition, we conducted a supplementary multivariate regression to isolate the key associations between the patterns and the characteristics of care recipients and primary family caregivers; this was carried out by multinomial logistic regression using the R3STEP command in Mplus, which accounts for classification error (Asparouhov & Muthén, 2014). Lastly, to test for associations of the identified patterns with caregiver depressive symptoms, distal outcome regressions were conducted using the Bolck–Croon–Hagenaars (BCH) command in Mplus, which accounts for classification error (Asparouhov & Muthén, 2021). The models also controlled for care recipients’ and primary family caregivers’ characteristics listed earlier (one respondent was excluded due to a missing response for employment status).

## Results

### Characteristics of Caregivers and Care Recipients

As reported in Table 1, most older adults received help from a primary family caregiver with socioemotional and other needs (89%) and ADLs and/or IADLs (86%), with slightly fewer receiving help with health and social care services use (68%). Fewer older adults received help from secondary caregivers for ADLs and IADLs (65%), health and social care services use (49%), and socioemotional and other needs (34%).

Care recipients had a mean age of 85 years and were mostly female (67%). Among them, 41% were currently married, and 55% had no formal education. On average, they had 4.6 chronic conditions, 2.3 ADL limitations, 3.9 IADL limitations, and an AD8 score for dementia symptoms of 3.6. Only 32% were using community care services.

Primary family caregivers had a mean age of 62 years and were mostly female (73%). Sixty percent of them were currently married, 69% had at least secondary education, 42% were working, 73% were the child of the care recipient, and 86% co-resided with their care recipient. They had an average of 3.5 siblings and a CES-D depressive symptom score of 4.6.

Care recipients had on average 2.3 caregivers (primary or secondary), and 48% had at least one MDW as a secondary caregiver (only three individuals had two MDWs). Table 2 provides a more detailed breakdown of the relationship between care recipients, primary family caregivers, and secondary caregivers. Most primary family caregivers were either the child (67%) or spouse (23%) of the care recipient. On the other hand, secondary caregivers were likely to be MDWs (38%), siblings or siblings-in-law (35%), or children (16%) of the primary family caregiver.

**Table 2.** Caregiving Relationships (%)

Variable	Relationship of primary family caregiver (*N* = 278) to care recipient	Relationship of secondary caregivers (*N* = 362) to primary family caregiver
Spouse	23.0	4.7
Child	66.6	16.0
Child-in-law	6.5	0.8
Sibling/sibling-in-law	2.2	34.8
Parent	—	3.3
Other relative	1.8	2.5
Migrant domestic worker	—	37.9

*Note*: Percentages of each type of secondary caregiver were calculated as a proportion of all secondary caregivers (*N* = 362).

### Family Caregiving Task-Sharing Patterns

**Figure 1.**
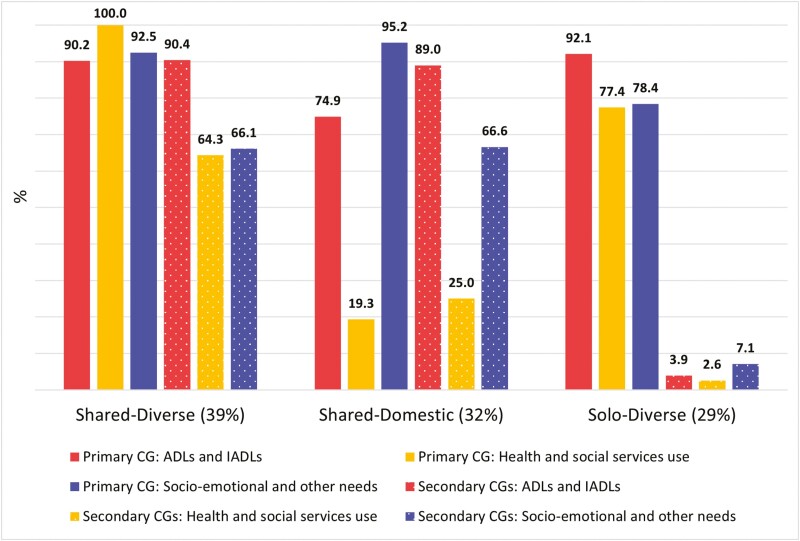
Family caregiving task-sharing patterns in latent class analysis. Data from 277 older adults and their caregivers (CGs). ADLs = activities of daily living; IADLs = instrumental ADLs.

The AIC and adjusted BIC were lowest for the three-class model, and the VLMR and BLRT were statistically significant (*p*-value < .05) for the three-class model but not for the four-class model (Supplementary Table 1). On the other hand, the cmP and entropy favored the two-class model. We therefore compared the caregiving task-sharing patterns between the two-class and three-class models (Supplementary Table 2). The three-class model provided greater analytic value, revealing a unique pattern where both the primary and secondary caregivers were unlikely to assist with health and social services use. Considering the theoretical and practical utility of these findings (Nylund-Gibson et al., 2023), we opted for the three-class model (Figure 1).

In the first pattern, both the primary and secondary caregivers were very likely to be involved in all three types of caregiving tasks, hence it was labeled “Shared-Diverse” (39%). In the second pattern, both the primary and secondary caregivers were very likely to be involved in assistance with ADLs and IADLs, as well as socioemotional and other needs, but not with health and social care services use; hence, it was labeled “Shared-Domestic” (32%). In the third pattern, only the primary family caregiver was very likely to be involved in all three types of caregiving tasks, hence it was labeled “Solo-Diverse” (29%).

Regarding the descriptive characteristics of the patterns (Table 3), significant differences were observed for care recipients’ gender, marital status, and functional limitations. Care recipients with the “Shared-Domestic” pattern were more likely to be female, single, and had more ADL and IADL limitations compared with those with other patterns.

**Table 3.** Care Recipient’s and Primary Family Caregiver’s Characteristics by Caregiving Task-Sharing Patterns

Variable	Pattern 1		Pattern 2		Pattern 3		*p*
	Shared-diverse (*n = *119)		Shared-domestic (*n = *74)		Solo-diverse (*n = *84)		
	%	Mean (*SD*)	%	Mean (*SD*)	%	Mean (*SD*)	
*Care recipient’s characteristics*							
Age		86.1 (5.5)		85.2 (5.4)		84.5 (5.1)	.105
Male	40.3		17.6		36.9		**.003**
Married, currently	47.1		24.3		45.2		**.004**
Education		1.7 (1.1)		1.6 (1.0)		1.8 (0.9)	.330
Chronic conditions		4.8 (2.4)		4.5 (2.9)		4.4 (2.3)	.616
ADL limitations		2.5 (2.5)		2.7 (2.3)		1.8 (2.1)	**.042**
IADL limitations		3.9 (1.9)		4.5 (2.0)		3.5 (1.8)	**.006**
AD8 score		3.9 (2.9)		3.8 (2.6)		3.0 (2.9)	.063
Use of community care services	37.8		25.7		28.6		.160
*Primary family caregiver’s characteristics*							
Age		60.4 (11.7)		60.3 (11.3)		64.7 (12.5)	**.021**
Male	23.5		27.0		31.0		.499
Married, currently	55.5		68.9		59.5		.175
Education		3.7 (1.5)		3.4 (1.5)		2.8 (1.4)	**<.001**
Employment status	50.4		44.6		27.7		**.005**
Child of care recipient	78.2		79.7		60.7		**.007**
Co-residing with care recipient	81.5		81.1		97.6		**.001**
Number of siblings		3.7 (2.1)		3.5 (2.0)		3.2 (2.2)	.208
*Caregiving context*							
Migrant domestic worker helps with caregiving	68.9		68.9		1.2		**<.001**
Total informal caregivers		2.8 (0.9)		2.6 (1.1)		1.3 (1.0)	**<.001**

*Notes*: *N* = 277. Class membership based on most likely class. AD8 = Eight-item Informant Interview to Differentiate Aging and Dementia; ADLs = activities of daily living; IADLs = Instrumental ADLs; SD = standard deviation. Bold *p*-values indicate significant differences between patterns (with ANOVA for continuous variables and chi-square for categorical variables).

A stark contrast was observed in the context of the “Solo-Diverse” pattern, where care recipients were the healthiest, having fewer ADL and IADL limitations and a lower AD8 score compared to the other patterns, but the primary family caregivers were the most vulnerable, being older, having lower education, and being less likely to be employed. Primary family caregivers in this pattern were also less likely to be a child (more likely to be the spouse) of the care recipient and highly likely to co-reside with the care recipient.

To further explore the independent contributing factors of the “Solo-Diverse” pattern, we conducted a multinomial logistic regression predicting the patterns by care-recipient and primary family caregiver characteristics (Supplementary Table 3). Primary family caregivers in the “Solo-Diverse” pattern were less likely to be better educated (relative risk ratio [RRR] = 0.56, 95% confidence interval [CI] = 0.37–0.86) and employed (RRR = 0.33, CI = 0.12–0.92) than those in the “Shared-Diverse” pattern, after controlling for covariates.

### Caregiver Depressive Symptoms by Patterns

In the distal outcome regression predicting depressive symptoms by the patterns (Table 4), compared with primary family caregivers in the “Shared-Diverse” reference pattern, those in the “Solo-Diverse” pattern had higher depressive symptoms (coefficient = 2.01; *p* = .021). Primary family caregivers in the “Shared-Domestic” pattern did not show significantly different outcomes compared with those in the “Shared-Diverse” pattern. For ease of interpretation, the regressions were repeated with a different reference pattern (Column 2); the results were consistent with the finding that primary family caregivers in the “Solo-Diverse” pattern had worse mental health than those in the other two patterns. The regressions controlled for covariates, for which coefficients are reported in Supplementary Table 4.

**Table 4.** Distal Outcome Regression Predicting Depressive Symptoms by Caregiving Task-Sharing Patterns

Caregiving task-sharing pattern	Main model		Alternative reference	
	Coefficient	[95% CI]	Coefficient	[95% CI]
Shared-Diverse	(Reference)		−0.89	[−2.62, 0.83]
Shared-Domestic	0.89	[−0.83, 2.62]	(Reference)	
Solo-Diverse	2.01*	[0.31, 3.72]	1.12	[−0.49, 2.73]

*Notes*: Observations = 276. Distal outcome regression conducted using the BCH command in Mplus. Model adjusted for care-recipient characteristics (age, gender, marital status, education, number of chronic conditions, number of basic and instrumental Activities of Daily Living limitations, Eight-item Informant Interview to Differentiate Aging and Dementia score, use of community care services) and primary family caregiver’s characteristics (age, gender, marital status, education, employment, child of care recipient, co-residence with care recipient, and number of siblings). BCH = Bolck–Croon–Hagenaars; CI = confidence interval.

*p < .05.

## Discussion

This study set out to understand how different caregiving tasks are shared among multiple caregivers, what caregiver and care-recipient characteristics are associated with the caregiving task-sharing patterns, and whether the way that caregiving is shared (or not) is related to the primary family caregiver’s depressive symptoms. These aims are important because contemporary research often focuses on the primary caregiver, and less is known about secondary caregivers, despite their prevalence in many contexts. Applying LCA, we found three distinct caregiving task-sharing patterns, labeled “Shared-Diverse,” “Shared-Domestic,” and “Solo-Diverse.” Caregivers in the “Solo-Diverse” pattern had higher depressive symptoms than primary family caregivers who had secondary caregivers sharing caregiving duties (“Shared-Diverse”). The study makes several key contributions, which we discuss with reference to their implications for research, policy, and practice.

First, we found it common in Singapore for caregiving tasks for older adults to be shared between multiple persons. The “Shared-Diverse” and “Shared-Domestic” patterns accounted for 71% of the membership, and the mean number of caregivers per care recipient was 2.3, suggesting that most primary family caregivers received support from at least one other person. Given Singapore’s historic notions of filial piety (Yeh, 2022), the limited coverage of its social long-term care system (Fong & Borowski, 2022), and the incentives provided to hire MDWs (Rozario & Hong, 2019), it is not surprising that older adults often receive help from multiple family caregivers and/or MDWs. This contrasts with the situation in the United States, where 51% of older adults are estimated to have a sole caregiver (Hu et al., 2023). Nevertheless, we note that there may be within-country differences—for example, collaborative caregiving is more common for Black and Hispanic care recipients and those with dementia (Ellis et al., 2023; Spillman et al., 2020).

Considering these findings, future research in global contexts should give attention to the roles of secondary caregivers and the sharing of caregiving tasks with persons beyond the nuclear family. Practitioners such as social workers should explicitly consider the presence and contributions of secondary caregivers when addressing the needs of primary family caregivers and care-recipient dyads, as discordant care coordination can be detrimental to caregiver outcomes (Xu et al., 2021). Caregivers who work together to care for the same person may need to develop stronger task-sharing skills (Spillman et al., 2020).

In addition, we found that secondary caregivers in our study were most likely to be MDWs, siblings, or siblings-in-law of the primary family caregiver. Among the recent literature that we reviewed on caregiving networks in the United States (Ali et al., 2022; Ellis et al., 2023; Liang et al., 2022; Spillman et al., 2020; Svec et al., 2024), only one study described the identity of a small sample of 30 secondary caregivers (Song et al., 2023). Future research on shared caregiving can add value by asking who these secondary caregivers are and what their relationship is to the primary caregiver, with implications for how they support and coordinate care.

Second, the study identified correlates of pattern membership. Care recipients in the “Shared-Domestic” pattern were more likely to be female and/or single and did not receive much assistance for health and social service needs. Some possible reasons, though not explored in the current study, could be that single or widowed female older adults are potentially less in need of health and social services, have less access to such services, are less willing to use such services or require less assistance to use these services. Further investigation, especially of a qualitative nature, should seek to understand gender differences in older adults’ service use patterns and need for assistance with access to services.

Third, we showed that caregivers in the “Solo-Diverse” pattern were more likely to display higher depressive symptoms compared with those in the “Shared-Diverse” pattern, corroborating prior literature on the stress of caregiving (Pearlin et al., 1990; Schulz et al., 2020). This was despite their care recipients being the healthiest among the three groups, pointing to the highly burdensome nature of solo caregiving regardless of care needs. Solo caregivers may be at high risk of mental health issues and should be the target of policy interventions such as respite care that allows them to take a break and participate in social activities. On the other hand, primary family caregivers who were supported by at least one secondary caregiver had lower depressive symptoms relative to solo caregivers; this could be partly attributed to “care domain overlap” (Liang et al., 2022), as the secondary caregivers in both the “Shared-Diverse” and “Shared-Domestic” patterns were largely helping with the same tasks as the primary family caregiver.

Our supplementary multivariate analysis showed that lower education and lack of employment were the factors that distinguished the “Solo-Diverse” caregivers after adjusting for covariates. These findings complement recent research on the positive role of support from secondary caregivers on the primary caregiver’s work strain (Svec et al., 2024), by highlighting the opposite scenario—that solo caregivers may struggle to balance informal caregiving and paid work. Future studies should investigate whether less educated caregivers experience difficulties coordinating care with others and whether their caregiving tasks are an obstacle to employment—which could lead to a lack of financial security in their own old age. Although caregiving may be rewarding at times, heavy caregiving demands if not shared are likely to take their toll on the primary—and only—caregiver (Peacock et al., 2010; Sung et al., 2023). Ultimately, this underscores the importance of sharing caregiving tasks and the need for further research in this area.

### Limitations

First, the data used is cross-sectional and does not show how caregiving task-sharing patterns change over the caregiving journey. This also precludes the use of more sophisticated methods that would support causal links between the patterns and caregiver depression. The study was also unable to explore more deeply the underlying or historical reasons for specific patterns; for example, whether someone became a solo caregiver because their siblings were unable to share caregiving responsibilities due to being overseas or other limitations. Second, the sample size is slightly smaller than what is typically recommended for LCA; nevertheless, a smaller sample size can still be acceptable if the latent classes are well-separated and theoretically meaningful (Nylund-Gibson & Choi, 2018). Third, the identification of one family member as a “primary” caregiver may gloss over the complexities of shared care, given that some caregivers consider themselves to have “equal” contributions (Hu et al., 2023; NAC & AARP, 2020), whereas more than one “primary” caregiver is possible in different contexts (Marcum et al., 2020). The reliance on the identified primary family caregiver to report on the caregiving tasks of other caregivers may also underestimate shared caregiving in some instances.

## Conclusion

The study contributes to our understanding of caregiving tasks and how they may be shared among multiple caregivers. The three identified caregiving task-sharing patterns, “Shared-Diverse,” “Shared-Domestic,” and “Solo-Diverse,” provide valuable insights into how caregiving happens and deserve further investigation as to the driving reasons behind each type of pattern. Policy-makers and practitioners need to be aware that these three groups may have different needs. For example, the roles of secondary caregivers have been overlooked in prior research, and future work should pay attention to their caregiving needs or burdens. Solo family caregivers, who are more likely to have poor mental health, maybe in dire need of support. Our findings underline the need for greater attention on how caregiving tasks may be distributed within and without the family, and how these patterns are influenced by cultural and institutional factors, with particular attention to societies with familial values.

## Supplementary Material

gbae186_suppl_Supplementary_Tables_S1-S4_Figure_S1

## Data Availability

The data are not publicly available; however, they can be made available upon request, subject to permission from the Centre for Ageing Research and Education, Duke-NUS Medical School. The survey was not preregistered.
