# Supplementary material for: Sharing in Caring: Family Caregiving Task-Sharing Patterns for Older Adults in Singapore
Source: J Gerontol B Psychol Sci Soc Sci. 2024 Nov 13;80(1):gbae186. doi: 10.1093/geronb/gbae186 (PMC11697107; doi:10.1093/geronb/gbae186)
Supplement: gbae186_suppl_Supplementary_Tables_S1-S4_Figure_S1 [file gbae186_suppl_supplementary_tables_s1-s4_figure_s1.docx]

***The Journals of Gerontology, Series B: Psychological Sciences and Social Sciences* Supplementary Material: Lim-Soh et al. Sharing in caring: Family caregiving task sharing patterns for older adults in Singapore**

**Supplementary Table 1**

*Class enumeration for Latent Class Analysis*

| Classes | Parameters | LL | AIC | BIC | aBIC | BLRT | VLMR | BF | cmP | LALCPP | Entropy |
| --- | --- | --- | --- | --- | --- | --- | --- | --- | --- | --- | --- |
| 1 | 6 | −895 | 1,803 | 1,825 | 1,806 | NA | NA | 0.0 | <.001 | NA | NA |
| 2 | 13 | −838 | 1,702 | **1,749** | 1,708 | <.001 | <.001 | >100 | 1.00 | .94 | .85 |
| 3 | 20 | −826 | **1,692** | 1,764 | **1,701** | **<.001** | **0.05** | >100 | <.001 | .84 | .71 |
| 4 | 27 | −820 | 1,694 | 1,792 | 1,706 | 0.19 | 0.16 | >100 | <.001 | .75 | .81 |
| 5 | 34 | −813 | 1,694 | 1,817 | 1,709 | 0.29 | 0.16 | >100 | <.001 | .90 | .88 |
| 6 | 41 | −809 | 1,699 | 1,848 | 1,718 | 0.50 | 0.12 | NA | <.001 | .79 | .86 |

*Note*. LL = Log likelihood, AIC = Akaike information criterion, BIC = Bayesian information criterion, aBIC = sample size adjusted BIC, BLRT = bootstrapped likelihood ratio test p-value, VLMR = Vuong-Lo-Mendell-Rubin adjusted likelihood ratio test p-value, BF = Bayes factor, cmP = approximate correct model probability, LALCPP = lowest average latent class posterior probability.

**Supplementary Table 2**

*Comparing 2 and 3-Class Solutions from Latent Class Analysis*

|  | **Pattern 1** | **Pattern 2** | **Pattern 3** |
| --- | --- | --- | --- |
| **2-class solution** |  |  |  |
| Primary CG: ADLs and IADLs (%) | 83.9 | 91.0 | - |
| Primary CG: Health and social services use (%) | 64.6 | 75.6 | - |
| Primary CG: Socio-emotional and other needs (%) | 93.7 | 77.9 | - |
| Secondary CGs: ADLs and IADLs (%) | 90.1 | 0.0 | - |
| Secondary CGs: Health and social services use (%) | 45.9 | 3.1 | - |
| Secondary CGs: Socio-emotional and other needs (%) | 65.6 | 6.9 | - |
| Membership (%) | 72.1 | 27.9 | - |
| **3-class solution** |  |  |  |
| Primary CG: ADLs and IADLs (%) | 90.2 | 74.9 | 92.1 |
| Primary CG: Health and social services use (%) | 100.0 | 19.3 | 77.4 |
| Primary CG: Socio-emotional and other needs (%) | 92.5 | 95.2 | 78.4 |
| Secondary CGs: ADLs and IADLs (%) | 90.4 | 89.0 | 3.9 |
| Secondary CGs: Health and social services use (%) | 64.3 | 25.0 | 2.6 |
| Secondary CGs: Socio-emotional and other needs (%) | 66.1 | 66.6 | 7.1 |
| Membership (%) | 38.9 | 32.2 | 28.8 |

*Note.* CG = caregiver, ADLs = Activities of Daily Living, IADLs = Instrumental ADLs.

**Supplementary Table 3**

*Multinomial Logistic Regression Predicting Caregiving Task Sharing Patterns*

| Variables | **Pattern 1** (39%) | | **Pattern 2** (32%) | | **Pattern 3** (29%) | |
| --- | --- | --- | --- | --- | --- | --- |
|  | *Shared-Diverse* | | *Shared-Domestic* | | *Solo-Diverse* | |
|  | RRR | [95% CI] | RRR | [95% CI] | RRR | [95% CI] |
| **Care recipient’s characteristics** |  |  |  |  |  |  |
| Age | (ref.) |  | 0.92 | [0.81, 1.04] | 0.94 | [0.85, 1.03] |
| Male | (ref.) |  | 0.27 | [0.07, 1.07] | 0.62 | [0.18, 2.20] |
| Married | (ref.) |  | 0.22 | [0.04, 1.09] | 0.39 | [0.13, 1.19] |
| Education | (ref.) |  | 0.99 | [0.56, 1.77] | 1.31 | [0.69, 2.49] |
| Chronic conditions | (ref.) |  | 0.93 | [0.76, 1.15] | 1.00 | [0.81, 1.23] |
| ADL and IADL limitations | (ref.) |  | 1.13 | [0.97, 1.32] | 0.92 | [0.80, 1.07] |
| AD8 score | (ref.) |  | 1.01 | [0.83, 1.22] | 0.89 | [0.75, 1.07] |
| Use of community care services | (ref.) |  | 0.46 | [0.14, 1.47] | 1.05 | [0.39, 2.80] |
| **Primary caregiver’s characteristics** |  |  |  |  |  |  |
| Age | (ref.) |  | 0.99 | [0.94, 1.04] | 0.99 | [0.94, 1.04] |
| Male | (ref.) |  | 1.15 | [0.34, 3.96] | 1.36 | [0.49, 3.77] |
| Married | (ref.) |  | 2.89 | [0.91, 9.12] | 0.81 | [0.28, 2.37] |
| Education | (ref.) |  | 0.78 | [0.52, 1.19] | 0.56** | [0.37, 0.86] |
| Working | (ref.) |  | 0.58 | [0.20, 1.73] | 0.33* | [0.12, 0.92] |
| Child of care recipient | (ref.) |  | 0.78 | [0.18, 3.37] | 0.98 | [0.24, 4.01] |
| Co-residing with care recipient | (ref.) |  | 0.71 | [0.12, 4.11] | 19.6 | [0.16, 2444.65] |
| Number of siblings | (ref.) |  | 0.92 | [0.75, 1.12] | 0.82 | [0.65, 1.05] |

*Note.* Regression conducted using the R3STEP command in Mplus. ADLs = Activities of Daily Living, IADLs = Instrumental ADLs, RRR = relative risk ratio, CI = confidence interval. Constants not shown.

* p < .05. ** p < .01.

**Supplementary Table 4**

*Distal Outcome Regression Predicting Primary Family Caregiver Depressive Symptoms by Caregiving Task Sharing Patterns (With Covariates)*

|  | Coefficient | [95% CI] |
| --- | --- | --- |
| **Caregiving pattern** |  |  |
| Shared-Diverse | (reference) |  |
| Shared-Domestic | 0.89 | [-0.83, 2.62] |
| Solo-Diverse | 2.01* | [0.31, 3.72] |
| **Care recipient’s characteristics** |  |  |
| Age | 0.02 | [-0.09, 0.14] |
| Male | 1.68* | [0.28, 3.07] |
| Married | 0.39 | [-0.97, 1.75] |
| Education | 0.34 | [-0.28, 0.95] |
| Chronic conditions | 0.19 | [-0.03, 0.42] |
| ADL and IADL limitations | 0.15* | [0.02, 0.29] |
| AD8 score | 0.18 | [-0.01, 0.37] |
| Use of community care services | 0.42 | [-0.73, 1.56] |
| **Primary caregiver’s characteristics** |  |  |
| Age | -0.05 | [-0.11, 0.02] |
| Male | 0.97 | [-0.18, 2.11] |
| Married | -1.10 | [-2.26, 0.06] |
| Education | -0.24 | [-0.65, 0.17] |
| Working | 0.04 | [-1.07, 1.15] |
| Child of care recipient | 0.03 | [-1.91, 1.97] |
| Co-residing with care recipient | -0.01 | [-1.59, 1.57] |
| Number of siblings | -0.03 | [-0.25, 0.19] |
| Observations | 276 |  |

*Note*. Distal outcome regression conducted using the BCH command in Mplus. ADLs = Activities of Daily Living, IADLs = Instrumental ADLs, AD8 = Eight-item Informant Interview to Differentiate Aging and Dementia. * p < .05.

**Supplementary Figure 1**

*Caregiving Task Sharing Patterns in Latent Class Analysis (Monochrome)*

*Note.* Data from 277 older adults and their caregivers (CGs). ADLs = Activities of Daily Living, IADLs = Instrumental ADLs.

*Alt text.* Bar graph showing three identified family caregiving task sharing patterns in Latent Class Analysis, named ‘Shared-Diverse’, ‘Shared-Domestic’, and ‘Solo-Diverse’.
